# Inhibitory Effects of Polyphenol- and Flavonoid-Enriched Rice Seed Extract on Melanogenesis in Melan-a Cells via MAPK Signaling-Mediated MITF Downregulation

**DOI:** 10.3390/ijms241411841

**Published:** 2023-07-24

**Authors:** Chaiwat Monmai, Jin-Suk Kim, Joong Hyoun Chin, Sanghyun Lee, So-Hyeon Baek

**Affiliations:** 1 Department of Agricultural Life Science, Sunchon National University, Suncheon 59722, Republic of Korea; bbuayy@gmail.com (C.M.); kimjs6911@naver.com (J.-S.K.); 2 Department of Integrative Biological Sciences and Industry, Sejong University, Seoul 05006, Republic of Korea; jhchin@sejong.ac.kr; 3 Department of Plant Science and Technology, Chung-Ang University, Anseong 17546, Republic of Korea; slee@cau.ac.kr

**Keywords:** polyphenols, flavonoids, antioxidant, anti-melanogenic, melanogenesis, MITF, tyrosinase activity, MAPK pathway, PI3K/Akt pathway

## Abstract

Melanin production is an important process that prevents the host skin from harmful ultraviolet radiation; however, an overproduction of melanin results in skin diseases. In the present study, we determined the antioxidative and anti-melanogenic activities of polyphenol- and flavonoid-enriched rice seed extracts in melan-a cells. The polyphenol and flavonoid content of Hopum (HP) and Sebok (SB) rice seed extracts was measured. The antioxidant capacity was determined using the ABTS radical scavenging method. SB contained high amounts of polyphenols and flavonoids, which significantly increased antioxidative activity compared with HP. Various concentrations of these extracts were evaluated in a cytotoxicity using melan-a cells. At 100 µg/mL, there was no significant difference for all treatments compared with untreated cells. Therefore, 100 µg/mL was selected as a concentration for the further experiments. SB significantly suppressed the phosphorylation/activation of p-38 MAPK, increased the expression of phosphorylated ERK 1/2 and Akt, and downregulated the microphthalmia-associated transcription factor (MITF). This resulted in decreased levels of tyrosinase and tyrosinase-related protein-1 and -2. These results indicate the potential of polyphenol- and flavonoid-enriched rice seed as a treatment for hyperpigmentation.

## 1. Introduction

Human skin is the largest organ in the body; it protects the host from various internal and external harmful, such as microorganisms, radiation, noxious substances, and body water loss [1]. Melanin production is a defense mechanism of the skin tissue that provides protection against ultraviolet radiation [2]. Microphthalmia-associated transcription factor (MITF) is a transcription factor involved in melanin production that enhances the production of tyrosinase, tyrosinase-related protein (TRP)-1, and TRP-2 [3,4,5]. The expression of MITF is also associated with the phosphorylation (p) of mitogen-activated protein kinases (MAPKs) [6,7,8]. The activation of p-p38 MAPK and p-JNK and the suppression of p-ERK 1/2 promote the activity of MITF and melanogenic-related enzymes during melanogenesis [9,10,11]. The production of melanin protects the host skin from ultraviolet radiation; however, an overproduction and excessive accumulation of melanin in the skin tissue results in skin diseases, such as melasma, freckles, skin discolorations, dark skin patches, and post-inflammatory melanoderma [3,12,13,14].

Polyphenols are secondary metabolites found in vegetables (such as kale, broccoli, and carrots) [15,16], grains and seeds (such as oats, almonds, barley, and wheat) [17,18,19], fruits (such as berries, apple, and grapes) [20], and beverages (such as green tea, red wine, and coffee) [21,22,23]. They exhibit potential health benefits such as protection against ultraviolet radiation [24], antiviral [25], wound healing [26,27], anti-inflammatory, antimicrobial, antioxidant [27], and anti-melanogenic [28] properties. Flavonoids, the largest group of polyphenols, are natural secondary metabolites that contain a polyphenolic structure [29,30,31]. Flavonoids exert several biological health benefits, such as antioxidant [32], antiradical [33], antiviral [34], anti-inflammatory [35], and anti-melanogenic [2] activities.

Breeding is one method of developing crop cultivars for the purpose of increasing yield production, resistance to climate changes, pest protection, and disease resistance [36]. We demonstrated the successful introgression of the target QTLs, which resulted in higher levels of polyphenols and flavonoids compared with Hopum (Figure S1). In the present study, we quantified polyphenols and flavonoids in Sebok rice seeds and evaluated their antioxidant and anti-melanogenic activities, thereby providing a potential treatment for hyperpigmentation.

## 2. Results

### 2.1. Total Polyphenol and Flavonoid Contents of Sebok Rice Seed Extract

We determined the total polyphenol and total flavonoid contents of SB extracts. The SB extract contained 21.6 mg tannic acid equivalent (TAE)/1 g of extract and 14.1 mg quercetin equivalent (QE)/1 g of extract (Table 1). The total polyphenol and flavonoid correlation coefficients were r^2^ = 0.99, indicating that both analyses were highly accurate, which likely occurred because the lower values obtained from the analysis were expressed in units of glycolic acid as the standard, whereas direct measurements of the phenol absorbance values were expressed in standard units.

### 2.2. Antioxidant Activities of Sebok Rice Seed Extract

**Figure 1:**
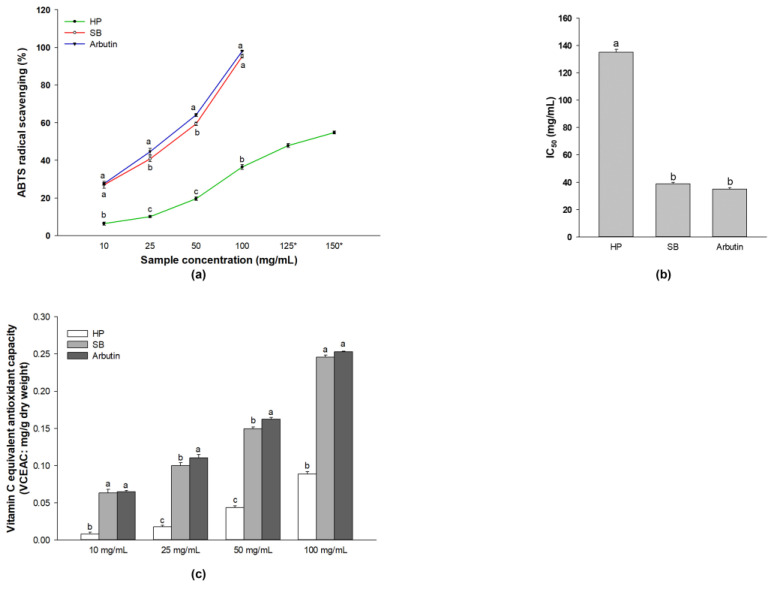
Antioxidant activity of polyphenol- and flavonoid-enriched rice seed extract. The effect on (**a**) ABTS radical scavenging, (**b**) IC_50_, and (**c**) vitamin C equivalent antioxidant capacity. Data are shown as the mean ± standard deviation. A one-way analysis of variance (ANOVA) followed by post hoc Duncan’s multiple range tests was used to determine the difference between treatments. Lowercase letters (a, b, and c) indicate significant differences at *p* < 0.05 among the HP, SB, and arbutin at the same concentration (where a > b > c).

The antioxidant activity of HP, SB, and arbutin was determined using the ABTS radical scavenging method (Figure 1a). Various concentrations (10, 25, 50, and 100 mg/mL) of extract were evaluated. The antioxidant activity increased in a concentration-dependent manner under all treatments. At a concentration of 100 mg/mL, SB exhibited the highest level of ABTS radical scavenging, which was identical to that of the positive control, arbutin.

The low sample concentration required to reduce the initial ABTS•+ concentration by 50% (IC_50_) indicates higher antioxidant activity. Figure 1b shows that SB and arbutin exhibited higher antioxidant activity than HP. Moreover, the antioxidant activity was expressed in terms of vitamin C (ascorbic acid) equivalent antioxidant capacity (VCEAC) (Figure 1c), which was determined using the standard curve of ABTS radical scavenging activity against ascorbic acid (vitamin C) concentration ranging from 0.39065 × 10^−2^ to 25 × 10^−2^ mg/mL (Figure S2).

### 2.3. Effect of Sebok Rice Seed Extract on Cell Viability

**Figure 2:**
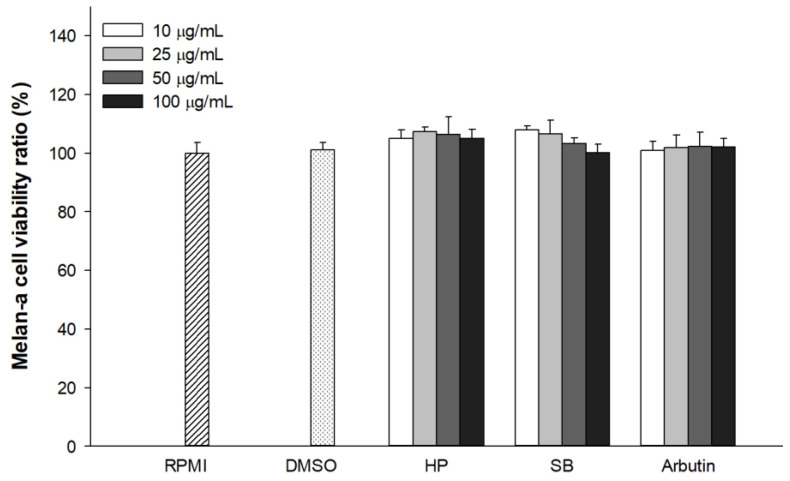
Effects of the polyphenols- and flavonoids-enriched rice seed extract on melan-a cell viability. DMSO concentration was 0.1%. Data are shown as mean ± standard deviation. Significant differences at *p* < 0.05.

Melan-a cells treated with the extracts and positive control (arbutin) at concentrations of 10–100 µg/mL (final concentration) were evaluated via the cytotoxicity assay using the EZ-CyTox cell viability solution. Figure 2 shows that there was no significant difference in melan-a cell viability compared with the untreated group (RPMI). Treatment with 0.1% DMSO did not show any cytotoxic or proliferative effect on melan-a cells compared with the RPMI group. These results indicate that treatment with 0.1% DMSO, HP, SB, or arbutin (up to 100 µg/mL) had no effect on melan-a cell viability compared with the RPMI group (untreated group).

### 2.4. Effect of Sebok Rice Seed Extract on Melanin Content and Melanin Excretion

**Figure 3:**
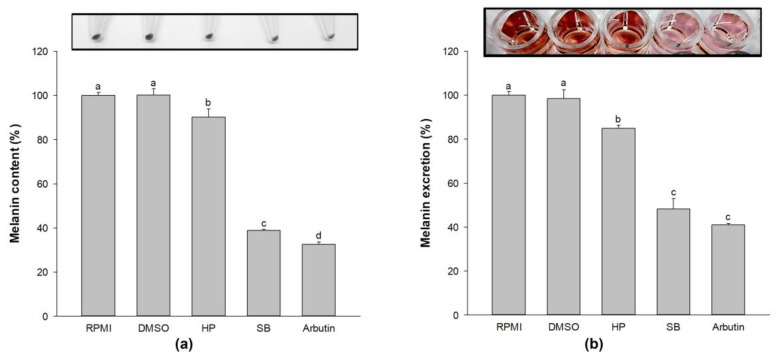
Effect of the polyphenol- and flavonoid-enriched rice seed extract on (**a**) melanin content and (**b**) melanin excretion. HP, SB, and arbutin concentrations were 100 µg/mL. The DMSO concentration was 0.1%. Data are shown as the mean ± standard deviation. A one-way ANOVA followed by post hoc Duncan’s multiple range tests was used to determine the difference between treatments. Lowercase letters (a, b, c, and d) indicate significant differences at *p* < 0.05 among the RPMI, DMSO, HP, SB, and arbutin (where a > b > c > d).

After 72 h incubation with 100 µg/mL extracts, the cells and culture media were collected separately. The cells were analyzed for melanin content, whereas the culture media was used to evaluate melanin excretion. The melanin content was significantly decreased in HP-, SB-, and arbutin-treated cells compared with that in the untreated group (*p* < 0.05, Figure 3a). Treatment with SB at a concentration of 100 µg/mL reduced the darkness of the pellet (visual observation) and significantly decreased cellular melanin content compared with the HP group (*p* < 0.05). In addition, 100 µg/mL of SB-treated cells significantly suppressed melanogenesis-associated melanin excretion (*p* < 0.05, Figure 3b) compared with the HP group. These results indicate that the treatment of SB at a concentration of 100 µg/mL resulted in a greater reduction in cellular melanin content and melanin excretion on melan-a cells compared with the normal rice seed extract (HP).

### 2.5. Effect of Sebok Rice Seed Extract on Cellular Tyrosinase Activity

**Figure 4:**
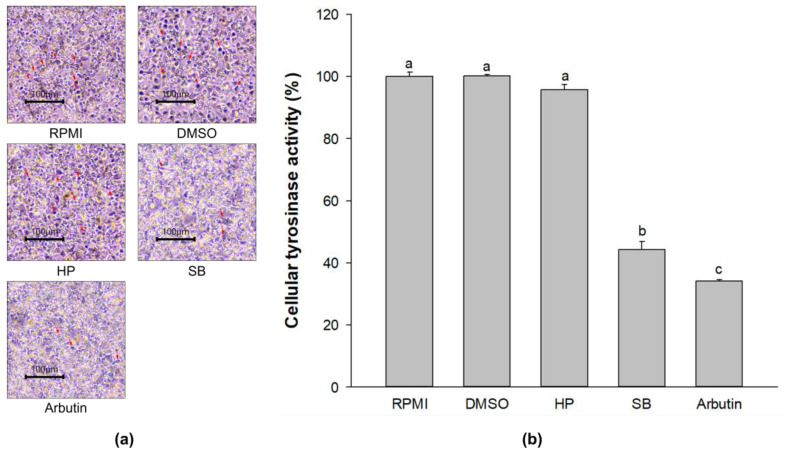
Effects of the polyphenol- and flavonoid-enriched rice seed extract on (**a**) L-DOPA staining and (**b**) cellular tyrosinase activity. The dark spots are the melanin-containing cells (red arrows). HP, SB, and arbutin concentrations were 100 µg/mL. The DMSO concentration was 0.1%. Data are shown as the mean ± standard deviation. A one-way ANOVA followed by post hoc Duncan’s multiple range tests was used to determine the difference between treatments. Lowercase letters (a, b, and c) indicate significant differences at *p* < 0.05 among the RPMI, DMSO, HP, SB, and arbutin (where a > b > c).

The dark spots indicate melanin-containing cells which mostly occurred in the RPMI-, DMSO-, and HP-treated cells (Figure 4a). Treatment with SB reduced the dark spots and showed lighter spots compared with treatment with HP. A decrease in the dark spots was confirmed by a reduction in cellular tyrosinase activity (Figure 4b). Compared with the RPMI group, no significant difference in cellular tyrosinase activity was observed in DMSO- and HP-treated cells (*p* < 0.05). Compared with the HP group, treatment of the cells with SB effectively reduced cellular tyrosinase activity from 95.70% ± 1.77% to 44.20% ± 2.70% (reduction by 53.81 ± 2.82% reducing). This finding indicates that SB significantly decreased cellular tyrosinase activity, which resulted in a reduction in the dark spots (melanin-containing cells) in melan-a cells.

### 2.6. Effect of Sebok Rice Seed Extract on Melanin-Containing Cells

**Figure 5:**
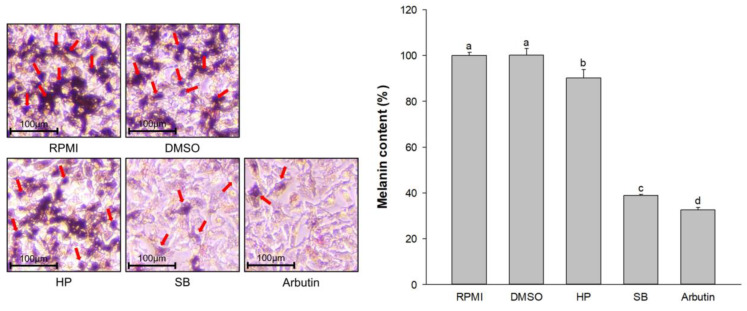
Effects of polyphenol- and flavonoid-enriched rice seed extract on melanin-containing cells. Melanin-containing melan-a cells were counted by observing 1000 cells under a microscope. Red arrows represent the melanin-containing melan-a cells. HP, SB, and arbutin concentrations were 100 µg/mL. The DMSO concentration was 0.1%. Data are shown as the mean ± standard deviation. A one-way ANOVA followed by post hoc Duncan’s multiple range tests was used to determine the difference between treatments. Lowercase letters (a, b, c, and d) indicate significant differences at *p* < 0.05 among the RPMI, DMSO, HP, SB, and arbutin (where a > b > c > d).

The number of melanin-containing cells was determined using the Fontana–Masson staining method. The dark spots indicated stained melanin in melan-a cells. There was no significant difference in the counts of melanin-containing cells between RPMI- and DMSO-treated cells (*p* < 0.05, Figure 5), which were 810 ± 6.7 and 809 ± 7.2 cells/1000 cells, respectively. Treatment with HP, SB, and arbutin (100 µg/mL) significantly decreased the number of melanin-containing cells compared with treatments with RPMI. Treatment with SB effectively reduced the number of melanin-containing cells (330 ± 12.7 cells/1000 cells) compared with treatments with HP (752 ± 13.5 cells/1000 cells), showing a similar number of melanin-containing cells compared with that in the positive control group (306 ± 13.1 cells/1000 cells).

### 2.7. Effect of Sebok Rice Seed Extract on the Morphological Appearance of Melan-a Cells

**Figure 6:**
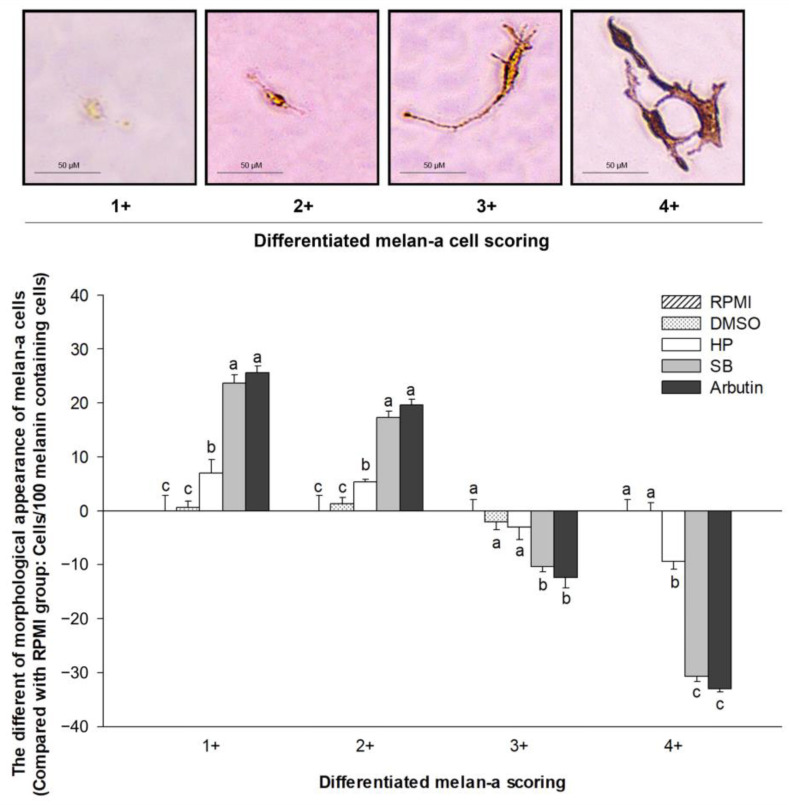
Effects of the polyphenol- and flavonoid-enriched rice seed extracts on the morphological appearance of melan-a cells. HP, SB, and arbutin concentrations were 100 µg/mL. The DMSO concentration was 0.1%. Data are shown as the mean ± standard deviation. A one-way ANOVA followed by post hoc Duncan’s multiple range tests was used to determine the difference between treatments. Lowercase letters (a, b, and c) indicate significant differences at *p* < 0.05 among the RPMI, DMSO, HP, SB, and arbutin (where a > b > c).

Melanin-containing cells were counted and categorized into four groups based on the differentiation score of 100 cells. For each differentiation score, the number of melanin-containing cells in each treatment group was compared with that in the RPMI group (Figure 6). Compared with the untreated group, treatment with HP significantly decreased the cell population with a differentiation score of 4+ and increased 1+ and 2+ populations (*p* < 0.05). SB treatment markedly reduced 3+ and 4+ populations and increased 1+ and 2+ populations compared with HP (*p* < 0.05). These results indicate that treatment with polyphenol- and flavonoid-enriched rice seed extract markedly reduced the melanin-containing population, size, and melanin contribution of melan-a cells.

### 2.8. Effect of Sebok Rice Seed Extract on Melanogenic-Related Gene Expression

**Figure 7:**
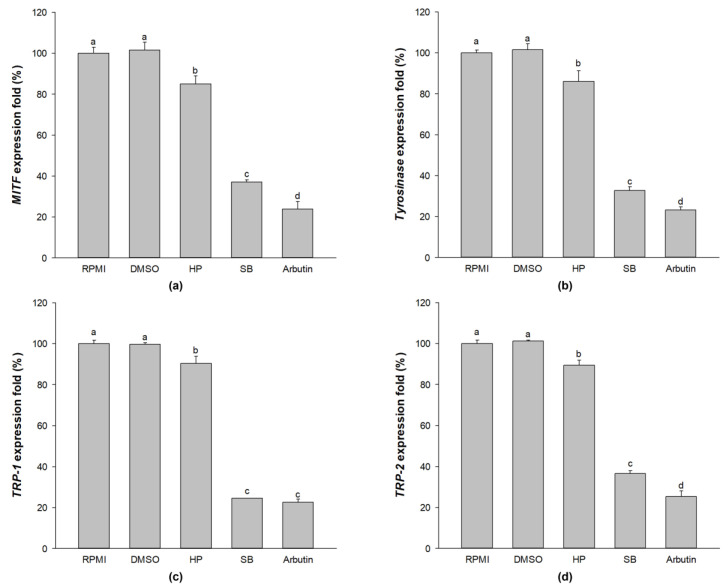
Effect of the polyphenols- and flavonoids-enriched rice seed extract on mRNA expression levels of melanogenesis mediators in melan-a cells. The expression levels of (**a**) MITF, (**b**) tyrosinase, (**c**), TRP-1, and (**d**) TRP-2. HP, SB, and arbutin concentrations were 100 µg/mL. DMSO concentration was 0.1%. Data are shown as mean ± standard deviation. A one-way ANOVA followed by post hoc Duncan’s multiple range tests was used to determine the difference between treatments. Lowercase letters (a, b, c, and d) indicate significant differences at *p* < 0.05 among the RPMI, DMSO, HP, SB, and arbutin (where a > b > c > d).

As shown in Figure 7, compared with the untreated group, treatment with SB significantly reduced the expression of melanogenic-related genes (MITF, tyrosinase, TRP-1, and TRP-2) (*p* < 0.05). In addition, treatment with SB exhibited significantly lower levels of MITF, tyrosinase, TRP-1, and TRP-2 compared with HP-treated cells. These results indicate that treatment with polyphenol- and flavonoid-enriched rice seed extract effectively inhibited the melanogenesis of melan-a cells.

### 2.9. Effect of Sebok Rice Seed Extract on Melanogenesis-Related Proteins

**Figure 8:**
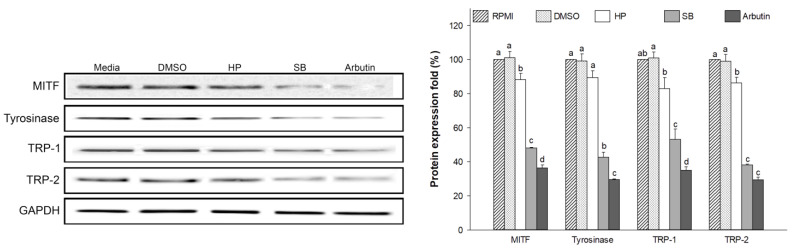
Effect of polyphenol- and flavonoid-enriched rice seed extract on melanogenesis-related protein expression. HP, SB, and arbutin concentrations were 100 µg/mL. The DMSO concentration was 0.1%. Data are shown as the mean ± standard deviation. A one-way ANOVA followed by post hoc Duncan’s multiple range tests was used to determine the difference between treatments. Lowercase letters (a, b, c, and d) indicate significant differences at *p* < 0.05 among the RPMI, DMSO, HP, SB, and arbutin (where a > b > c > d).

RPMI- and DMSO-treated cells exhibited the highest expression of MITF, which is a transcription factor that regulates TRP-1, TRP-2, and tyrosinase expression during melanogenesis. A high expression of MITF also resulted in a high expression of tyrosinase, TRP-1, and TRP-2 proteins (Figure 8). Compared with RPMI and HP treatments, treatment with 100 µg/mL SB downregulated the expression of MITF protein, which resulted in a reduced expression of tyrosinase, TRP-1, and TRP-2 [37]. These results indicate that SB treatment significantly inhibited melanogenesis by downregulating the expression of MITF, which resulted in decreased levels of tyrosinase, TRP-1, and TRP-2 in melan-a cells.

### 2.10. Effect of Sebok Rice Seed Extract on the MAPKs and PI3K/Akt Signaling Pathways

**Figure 9:**
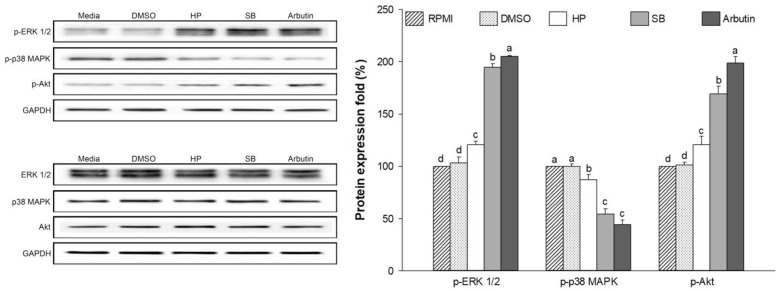
Effect of the polyphenol- and flavonoid-enriched rice seed extract on inflammatory related protein expression. The HP, SB, and arbutin concentrations were 100 µg/mL. The DMSO concentration was 0.1%. Data are shown as the mean ± standard deviation. A one-way ANOVA followed by post hoc Duncan’s multiple range tests were used to determine the difference between treatments. Lowercase letters (a, b, c, and d) indicate significant differences at *p* < 0.05 among the RPMI, DMSO, HP, SB, and arbutin (where, a > b > c > d).

The regulation of MITF is associated with ERK 1/2, p38 MAPK, and Akt signaling pathways [38]. Therefore, p-ERK 1/2, p38 MAPK, and Akt proteins were evaluated. As shown in Figure 9, treatment with SB significantly increased the expression of p-ERK 1/2 and p-Akt compared with RPMI or HP treatment (*p* < 0.05). However, compared with RPMI or HP treatment, treatment with SB markedly reduced the expression of p-p38 MAPK in SB-treated cells (*p* < 0.05). The regulation of these protein levels in SB-treated cells showed a similar trend as that in arbutin-treated cells.

## 3. Discussion

Oxidative stress is harmful to human health, as it can damage cell membranes and lipoproteins through lipid peroxidation [39], damaging proteins undergoing conformational modifications [40], damaging DNA [41], and promoting cancer development [42]. In the present study, we demonstrated that SB, which contains high levels of polyphenols and flavonoids, significantly promotes antioxidant activity by increasing ABTS radical scavenging ability at increased concentrations compared with HP (Figure 1). The antioxidant activity of SB for both ABTS radical scavenging (%) and VCEAC was remarkably correlated with polyphenol (Pearson’s correlation coefficient = 0.9091, *p* < 0.01) and flavonoid (Pearson’s correlation coefficient = 0.9783, *p* < 0.01) contents. Similarly, Guaita, M. and Bosso, A. [43] reported a correlation between the polyphenol content and antioxidant activity. Further, Asem, N. et al. [44] demonstrated a strong correlation of total phenolic and flavonoid contents with their antioxidant activity.

**Figure 10:**
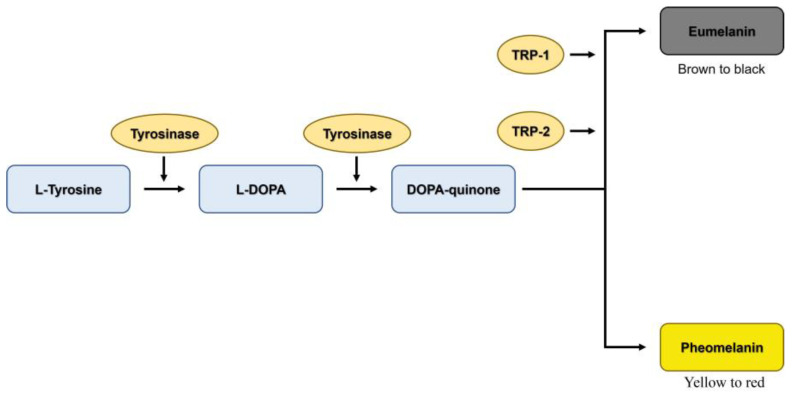
Simplified scheme of melanin synthesis during melanogenesis.

There are two melanogenesis pathways depending on the final form of melanin. The first form is pheomelanin, which shows yellow to red pigmentation, whereas the second form is eumelanin, which exhibits dark brown/black pigmentation [45]. Eumelanin is the most common form of melanin that protects the skin from ultraviolet radiation [46]. L-tyrosine is hydroxylated to L-dihydroxyphenylalanine (L-DOPA), which is further oxidized to DOPA-quinone by tyrosinase [47]. Eumelanin synthesis requires the two key enzymes of the melanogenesis pathway, TRP-2 and TRP-1 (Figure 10). Therefore, the reduction in the expression of tyrosinase, TRP-1, and TRP-2 results in decreased melanin production. Our results revealed that SB-treated cells significantly downregulate the expression of tyrosinase, TRP-1, and TRP-2 at mRNA (Figure 7b–d) and protein (Figure 8) levels. The polyphenol-enriched *Rosa rugosa* extract exerts similar effect as observed in the current study by reducing the mRNA and protein expression levels of MITF, tyrosinase, TRP-1, and TRP-2 in murine melanoma cells [48]. In addition, Shin, S. et al. [49] demonstrated that the colloidal gold green tea extract, which contains high levels of phenols and flavonoids, increased antioxidative and anti-melanogenic activities via the downregulation of MITF, tyrosinase, TRP-1, and TRP-2.

MITF is an important transcription factor in melanin synthesis that regulates the production of tyrosinase, TRP-1 and TRP-2 [3]. The expression of MITF is related to the phosphorylation of MAPK and PI3K/Akt-associated proteins [3,6,9]. The activation of p-p38 MAPK promotes MITF expression, which leads to the enhancement of melanogenesis [50]. Conversely, the activation of p-ERK 1/2 and p-Akt leads to the ubiquitination and degradation of MITF, resulting in the reduction in tyrosinase, TRP-1, and TRP-2 levels [51,52]. This finding indicates that the activation of p-p38 MAPK promotes melanogenesis by preventing MITF degradation, whereas the activation of p-ERK 1/2 and p-Akt enhances anti-melanogenesis by promoting MITF ubiquitination and degradation. The present study showed that SB treatment significantly downregulated the expression of p-p38 MAPK (melanogenesis promoter) and up-regulated the expression of p-ERK 1/2 and p-Akt (inducers of MITF degradation) when compared with HP treatment (Figure 10). The regulation of these proteins resulted in the decreased expression of MITF mRNA (Figure 7a) and protein (Figure 8). Similarly, Alam, M.B. et al. [53] reported that polyphenols extracted from *Heracleum moellendorffii* Hance induced the activation of p-ERK 1/2, which led to the degradation of MITF and suppression of tyrosinase, TRP-1, and TRP-2 levels. Moreover, Lee, S.-G. et al. [2] reported that flavonoid glycosides from *Limonium tetragonum* (Thunb.) exhibited anti-melanogenic activity by suppressing tyrosinase activity, TRP-1 expression, and TRP-2 expression in B16-F10 mouse melanoma cells.

The inhibition of melanogenesis by polyphenol- and flavonoid-enriched rice seed extract (SB) also affected the morphological appearance of melan-a cells by reducing the cellular melanin distribution and number of melanin-containing cells. This is consistent with that of the study by Yu, Q. and Fan, L. [54], which demonstrated the effect of asparagus polyphenol extract on B16F10 cell morphology. They revealed that treatment with a high concentration of polyphenols in murine melanoma cells extensively suppressed melanin production and decreased the melanin-containing cell size and number.

The antioxidative and anti-melanogenic activities of Sebok rice seed extract were determined through comparison of its parent, Hopum. SB significantly promoted the antioxidative effect by enhancing the ABTS radical scavenging activity. Activities related to melanin production were significantly inhibited in SB-treated cells via the downregulation of the expression of melanogenesis-related transcription factors and proteins, such as MITF, tyrosinase, TRP-1, and TRP-2. The difference between HP and SB is that SB contains polyphenols and flavonoids. Our results indicate that the high level of polyphenols and flavonoids content in SB may be attributed to the antioxidative and anti-melanogenic activities. At identical low concentrations (25 and 50 mg/mL), treatment with arbutin (positive control) induced stronger antioxidative activity than that with SB. However, no significant differences were noted in the antioxidative activity between treatments with SB and arbutin at 100 mg/mL. Interestingly, the anti-melanogenic activities of SB were similar to those of arbutin (positive control). Compared with treatment with arbutin, treatment with 100 μg/mL SB revealed similar inhibition effects on melanin excretion, number of melanin-containing cells, and the melan-a morphological appearance when compared with arbutin. Moreover, the antioxidative and anti-melanogenic activities were observed in the HP group. Based on these results, SB may exert antioxidative and anti-melanogenic activities through the cooperative effects of polyphenols and flavonoids and original compounds in rice.

## 4. Materials and Methods

### 4.1. Reagents and Materials

ABTS was purchased from Roche (Basel, Switzerland). Arbutin, potassium persulfate, 12-*O*-tetradecanoylphorbol-13-acetate (TPA), L-DOPA, Folin–Ciocalteu phenol reagent, NaOH, and formaldehyde were purchased from Sigma-Aldrich (St. Louis, MO, USA). Tannic acid and quercetin were obtained from the Natural Product Institute of Science and Technology (Anseong, Korea). RPMI-1640 medium and fetal bovine serum (FBS) were obtained from Gibco™ (Thermo Fisher Scientific, Inc., Waltham, MA, USA). The EZ-CyTox Cell Viability Kit was purchased from DoGenBio (Seoul, Republic of Korea). Penicillin/streptomycin (P/S) was obtained from Hyclone Laboratories, Inc (Logan, UT, USA). Triton X-100 was obtained from R&D systems, Inc. (Minneapolis, MN, USA). Bradford reagent was obtained from WELGENE, Inc. (Gyeongsangbuk-do, Republic of Korea). TRI reagent™ was obtained from Invitrogen (Waltham, MA, USA). The Power cDNA Synthesis Kit and RealMOD™ Green W^2^ 2× qPCR mix were purchased from Intron Biotechnology (Seongnam-si, Republic of Korea). The Fontana-Masson kit was obtained from BIOGNOST, Ltd. (Zagreb, Croatia). Radioimmunoprecipitation assay buffer (RIPA) was purchased from GeneAll Biotechnology (Seoul, Republic of Korea). The Protease Inhibitor Cocktail Kit 5 was purchased from Bio-Medical Science Co., Ltd. (Seoul, Republic of Korea). The primary antibodies against MITF (97800S), phosphorylated (p)-ERK 1/2 (4377S), p-p38 MAPK (4511S), p-Akt (9271S), and ERK 1/2 (4695S) were purchased from Cell Signaling (Danvers, MA, USA). The primary antibodies against tyrosinase (sc-20035), TRP-1 (sc-166857), TRP-2 (sc-74439), Akt (sc-5298), p38 MAPK (sc-7972), and GAPDH (sc-32233) were obtained from Santa Cruz Biotechnology (Dallas, TX, USA). Goat anti-rabbit IgG(H + L)-HRP (SA002-500) was obtained from GenDEPOT (Baker, TX, USA) and m-IgGκ BP-HRP (sc-516102) antibody was purchased from Santa Cruz Biotechnology (Dallas, TX, USA). Charity™ Western ECL substrate, ChemiDoc Imaging System, and the CFX Connect Real-Time PCR System were purchased from Bio-Rad (Hercules, CA, USA). A SpectraMax^®^ ABS Plus microplate reader was obtained from Molecular Devices (San Jose, CA, USA). An Epoch microplate reader was purchased BioTek, Winooski, VT, USA. The IM-3 series microscope was purchased from Optika (Bergamo, Italy). Statistix (version 8.1) was obtained from Statistix (Tallahassee, FL, USA).

### 4.2. Treatment Preparation

Hopum and Sebok rice seeds were produced on the research farm of Sejong University. They were unpeeled, ground into a fine powder, and extracted with 80% methanol as previously described [55]. Extracts were prepared in DMSO at concentrations of 10, 25, 50, and 100 mg/mL for the experiments.

Arbutin is a compound of hydroquinone and D-glucose [56]. Several studies have demonstrated the anti-melanogenic activities of arbutin, in which melanin production and hyperpigmentation are inhibited in α-MSH-stimulated B16 cells [57,58]. Melanin content and intracellular tyrosinase activity were also reduced in murine melanoma B16 cells [59]. Therefore, arbutin was used as a positive control in our experiments. Arbutin was prepared at concentrations of 10, 25, 50, and 100 mg/mL.

### 4.3. Total Polyphenol and Flavonoid Contents Determination

The total polyphenol content of SB was measured as described previously [60,61] with slight modifications in the calibration curve construction. Briefly, 60 μL of SB extract was mixed with 40 μL of 2 N Folin–Ciocalteu phenol reagent. Then, 100 μL of 7.5% sodium carbonate solution was added to the mixture, which was incubated for 30 min under dark conditions. The absorbance of the samples was measured using an Epoch microplate reader at 760 nm. Finally, a calibration curve was constructed using tannic acid as the reference [62] (Figure S3), and the total polyphenol content was quantified. The total flavonoid content of SB was analyzed using a previously described method [61] with slight modifications. Briefly, 100 μL of 1 mg/mL SB extract was mixed with 100 μL of 2% AlCl_3_. The solution was incubated for 10 min, and the absorbance was measured at 430 nm using an Epoch microplate reader. A calibration curve was constructed using quercetin as the standard (Figure S4), and the total flavonoid content was determined.

### 4.4. Antioxidant Activity Assay

The antioxidant activity of polyphenol- and flavonoid-enriched rice seed extract was determined by measuring ABTS radical scavenging ability (ABTS^•+^). The ABTS^+^ cation (ABTS^•+^) decolorization assay method was performed as previously described [63]. Briefly, ABTS^•+^ was prepared by mixing ABTS with potassium persulfate at final concentrations of 7.0 and 2.4 mM, respectively. The mixture was incubated at room temperature for 16 h under dark conditions. Then, it was then diluted in 100% ethanol to obtain an absorbance value of 0.70 ± 0.02 at 734 nm (ABTS^•+^ working solution). The extracts at different concentrations (10 µL) were incubated together with 1 mL of ABTS^•+^ working solution at room temperature for 7 min under dark conditions. Then, the absorbance of the samples was measured at 734 nm, and 10 µL of distilled water and 1 mL ABTS^•+^ working solution served as the control. The experiment was performed in triplicate. The ABTS radical scavenging ability (%) was calculated according to the following formula:

(1) $$\mathrm{ABTS}\;\mathrm{radical}\;\mathrm{scavenging}\;(\%)=\left[\frac{\mathrm{Absorbance}\;\mathrm{at}\;734\;\mathrm{nm}\;\mathrm{of}\;\mathrm{control}\;-\;\mathrm{Absorbance}\;\mathrm{at}\;734\;\mathrm{nm}\;\mathrm{of}\;\mathrm{sample}}{\mathrm{Absorbance}\;\mathrm{at}\;734\;\mathrm{nm}\;\mathrm{of}\;\mathrm{control}}\right]\;\times \;100,$$

Varying concentrations of ascorbic acid were measured using the ABTS radical scavenging assay, and a standard curve of ascorbic acid (X) against ABTS radical scavenging (Y) was constructed (Figure S2) to calculate the vitamin C (ascorbic acid) equivalent antioxidant capacity (VCEAC). VCEAC was assessed using the following formula:

(2) $$\mathrm{VCEAC}\;(\mathrm{mg}/g\;\mathrm{dry}\;\mathrm{weight})=\frac{Y\;-\;3.3284}{3.7463},$$

where Y represents the ABTS radical scavenging.

The sample concentration required to reduce the initial ABTS^•+^ concentration by 50% (IC_50_) was determined by plotting the percentage of scavenging (X) against various concentrations of the extracts (Y). The IC_50_ was calculated by substituting the value of X with 50 in the regression equation of Y = AX + B.

### 4.5. Viability Assay of Melan-a Cells

Melan-a cells were cultured in RPMI-1640 medium (10% FBS, 1% P/S, and 20 nM TPA supplementation) and incubated at 37°C under 5% CO_2_. Cells were seeded into a 96-well plate at a density of 2 × 10^4^ cells/well and incubated at 37 °C under 5% CO_2_. After 24 h, the culture medium was replaced with varying concentrations of the extracts (diluted in the culture medium). The cells were further incubated under the same conditions for 72 h. The culture medium was discarded, and 110 µL of EZ-CyTox (10-fold dilution in 1× PBS) was added into each well. The plate was incubated at 37 °C for 4 h. Then, 100 µL of the EZ-CyTox solution was transferred into a new 96-well plate, and the absorbance was measured at 450 nm using SpectraMax^®^ ABS Plus Microplate Reader. The melan-a cell viability ratio was determined according to the following formula:

(3) $$\mathrm{Melan}-a\;\mathrm{cell}\;\mathrm{viability}\;\mathrm{ratio}\;(\%)=\frac{A_{450}\mathrm{of}\;\mathrm{treatment}}{A_{450}\mathrm{of}\;\mathrm{control}}\;\times \;100,$$

where A_450_ represents the absorbance at 450 nm. The RPMI-treated cells (without treatment) group was used as a control.

### 4.6. Melanin Content and Melanin Excretion Assay

The cells were seeded into a 6-well plate at a density of 5 × 10^5^ cells/well and incubated at 37 °C under 5% CO_2_. After 24 h, the cells were treated with 100 µg/mL of the extracts. The plate was further incubated for 72 h at 37 °C under 5% CO_2_. The culture medium and cells were collected separately. For melanin excretion, the culture medium was centrifuged at 2000 rpm for 3 min, and the absorbance mas measured at 405 nm. The following formula was used for melanin excretion evaluation.

(4) $$\mathrm{Melanin}\;\mathrm{excretion}\;(\%)=\frac{\mathrm{Absorbance}\;\mathrm{at}\;405\;\mathrm{of}\;\mathrm{treatment}}{\mathrm{Absorbance}\;\mathrm{at}\;405\;\mathrm{of}\;\mathrm{control}}\;\times \;100,$$

The cultured cells were counted, and the cell number was adjusted to 1 × 10^5^ cells/treatment. The cells were disrupted with 1 N NaOH solution at 80 °C for 4 h. The absorbance of treatment and control groups was measured at 405 nm. The melanin content was estimated using the following formula:

(5) $$\mathrm{Melanin}\;\mathrm{content}\;(\%)=\frac{\mathrm{Absorbance}\;\mathrm{at}\;405\;\mathrm{of}\;\mathrm{treatment}}{\mathrm{Absorbance}\;\mathrm{at}\;405\;\mathrm{of}\;\mathrm{control}}\;\times \;100,$$

The experiment was conducted in triplicate independently. RPMI-treated cells were used as a control.

### 4.7. L-DOPA Staining and Cellular Tyrosinase Activity Assay

L-DOPA staining was performed as previously described [63,64]. Melan-a cells were seeded into a 96-well plate at a density of 2 × 10^4^ cells/well and incubated at 37 °C under 5% CO_2_ for 24 h. Then, the cells were treated with 100 µg/mL of extract and further incubated for 72 h. After washing with 1× PBS, the cells were fixed with 10% formaldehyde at room temperature for 20 min and washed with 1× PBS. Subsequently, 100 µL of 2 mg/mL L-DOPA was added into each well. Staining was performed at 37 °C for 3 h. The solution was removed, and the cells were washed twice with 1× PBS. After drying, the pigmentation of melan-a cells was observed and imaged using an IM-3 series microscope.

To measure tyrosinase activity, the treated cells were collected and lysed with 0.1 M sodium phosphate (pH 6.8) containing 1% Triton X-100 on ice for 30 min [63]. The tube was centrifuged for 30 min at 13,000 rpm and 4 °C. The supernatant was collected, and protein concentration was measured using the Bradford method. Furthermore, 40 µg of protein from each treatment was adjusted with lysis buffer in a total volume of 80 µL. L-DOPA at 2 mg/mL (20 µL) was added, the samples were incubated at 37 °C for 1 h, and the absorbance was measured at 475 nm. The following formula was used to determine cellular tyrosinase activity (%):

(6) $$\mathrm{Cellular}\;\mathrm{tyrosinase}\;\mathrm{activity}\;(\%)=\left[\frac{\mathrm{Absorbance}\;\mathrm{at}\;475\;\mathrm{nm}\;\mathrm{of}\;\mathrm{RPMI}\;\mathrm{group}\;-\;\mathrm{Absorbance}\;\mathrm{at}\;475\;\mathrm{nm}\;\mathrm{of}\;\mathrm{sample}}{\mathrm{Absorbance}\;\mathrm{at}\;475\;\mathrm{nm}\;\mathrm{of}\;\mathrm{RPMI}\;\mathrm{group}}\right]\;\times \;100,$$

### 4.8. Morphological Appearance Assay

After 72 h incubation, the treated cells were stained using a Fontana-Masson kit according to the manufacturer’s instructions. The stained cells were monitored and counted for a total of 1000 cells using an IM-3 series microscope. The number of melanin-containing cells was recorded. A total of 100 melanin-containing cells were randomly counted and categorized into 4 groups (1+, 2+, 3+, and 4+) according to their morphological appearance using a scoring system described by Rodboon, T. et al. [65] (Table S1).

### 4.9. RNA Extraction and Gene Expression Quantification

Total RNA was extracted from the treated cells using TRI reagent™ and dissolved in nuclease-free water. The concentration of the isolated RNA was measured, and the quality was determined using a SpectraMax^®^ ABS Plus Microplate Reader. The quality of the RNA was assessed by the ratio of the absorbance at 260 and 280 nm (A260:A280) and the absorbance at 260 and 230 nm (A260:A230), which was in the acceptable range (1.80–2.00). Total RNA (1000 ng) was synthesized using the Power cDNA Synthesis Kit. The qPCR reactions consisted of 0.375 µM of each primer (forward and reverse, Table 2) and 5 ng of template (synthesized cDNA) in a RealMOD™ Green W^2^ 2× qPCR Mix following the manufacturer’s instructions, and it was run on a CFX Connect Real-Time PCR system. The PCR conditions included 1 cycle at 95 °C for 10 min; 40 cycles of denaturation (95 °C for 20 s), annealing (60 °C for 20 s), and elongation (72 °C for 30 s); and 1 final elongation cycle at 72 °C for 5 min. The relative gene expression levels were determined using the CFX Connect Real-Time PCR program with glyceraldehyde 3-phosphate dehydrogenase (GAPDH) as a housekeeping gene.

### 4.10. Western Blot Assay

Treated cells were lysed in RIPA buffer containing 1× Protease Inhibitor Cocktail Kit 5 on ice for 30 min and centrifuged at 13,000 rpm for 30 min at 4 °C. The solution was transferred to a new microcentrifuge tube. Protein concentrations were determined using the Bradford method and compared with a bovine serum albumin standard curve. The protein was adjusted to 30 µg/treatment and subject to sodium dodecyl sulfate-polyacrylamide gel electrophoresis. The proteins were transferred onto a nitrocellulose membrane, which was blocked and incubated with primary antibodies specific to MITF, TRP-1, TRP-2, phosphorylation (p)-ERK 1/2, p-p38 MAPK, p-Akt, ERK 1/2, p38 MAPK, Akt, and GAPDH at 4 °C overnight. After washing, the membrane was incubated with a secondary antibody (Goat anti-rabbit IgG(H + L)-HRP or m-IgGκ BP-HRP) at room temperature for 2 h. The protein signals were detected using Clarity™ Western ECL Substrate. The protein signals were captured and quantified using the ChemiDoc Imaging System.

### 4.11. Statistical Analysis

The data are shown as means ± standard deviations. Statistical analyses were performed using Statistix (version 8.1; Statistix, Tallahassee, FL, USA). The data analysis included a one-way analysis of variance followed by post hoc Duncan’s multiple range tests. Differences between two groups were assessed using t-tests at a significance level of *p* < 0.05.

## 5. Conclusions

The present study demonstrated the potential effects of polyphenol- and flavonoid-enriched rice seed extract on antioxidant and anti-melanogenic activity. Sebok rice seed extract containing polyphenols and flavonoids exerted an antioxidant effect by enhancing ABTS radical scavenging ability. Polyphenol- and flavonoid-enriched rice seed extract downregulated the production of the melanogenesis-related transcription factor, MITF. In addition, this extract suppressed the production of p-p38 MAPK and increased the production of p-ERK 1/2 and p-Akt. These regulations led to a reduction in tyrosinase, TRP-1, and TRP-2 expression at mRNA and protein levels. Decreased melanogenesis resulted in the reduction in size and melanin dispersion in melan-a cells. These findings demonstrate that polyphenol- and flavonoid-enriched rice may be considered a novel agent for controlling hyperpigmentation.

## Figures and Tables

**Table 1:** Total polyphenol and total flavonoid contents contained in rice seed extract.

Extract	Total Polyphenols Content (mg TAE/g Extract)	Total Flavonoids Content (mg QE/g Extract)
HP	12.5 ± 0.6	3.1 ± 1.1
SB	21.6 ± 1.2	14.1 ± 1.5

**Table 2:** Forward and reverse primer sets used in this experiment.

Gene	Accession Number	Sequence (5′-3′)	Target Size (bp)
*MITF*	NM_001113198.2	Forward: AGC GTG TAT TTT CCC CAC AG Reverse: CCT TAG CTC GTT GCT GTT CC	239
*Tyrosinase*	D00131.1	Forward: CCA GAA GCC AAT GCA CCT AT Reverse: CCA GAT ACG ACT GGC CTT GT	193
*TRP-1*	NM_031202.3	Forward: TCT GGC CTC CAG TTA CCA AC Reverse: TCA GTG AGG AGA GGC TGG TT	223
*TRp-2*	X63349.1	Forward: ACC CTG TGT TTG TGG TCC TC Reverse: GTT GCT CTG CGG TTA GGA AG	186
*GAPDH*	NM_001289726.2	Forward: AAC TTT GGC ATT GTG GAA GG Reverse: ACA CAT TGG GGG TAG GAA CA	223

## Data Availability

The data are contained within the manuscript.

## Supplementary Materials

The supporting information can be downloaded at: https://www.mdpi.com/article/10.3390/ijms241411841/s1.
