# Supplementary material for: Inhibitory Effects of Polyphenol- and Flavonoid-Enriched Rice Seed Extract on Melanogenesis in Melan-a Cells via MAPK Signaling-Mediated MITF Downregulation
Source: Int J Mol Sci. 2023 Jul 24;24(14):11841. doi: 10.3390/ijms241411841 (PMC10380342; doi:10.3390/ijms241411841)
Supplement: Supplementary file 1 [file ijms-24-11841-s001.zip › ijms-2506977-supplementary.pdf]

Supplementary Materials

Figure S1. Schematic of breeding of the rice variety, 'Sebok'.

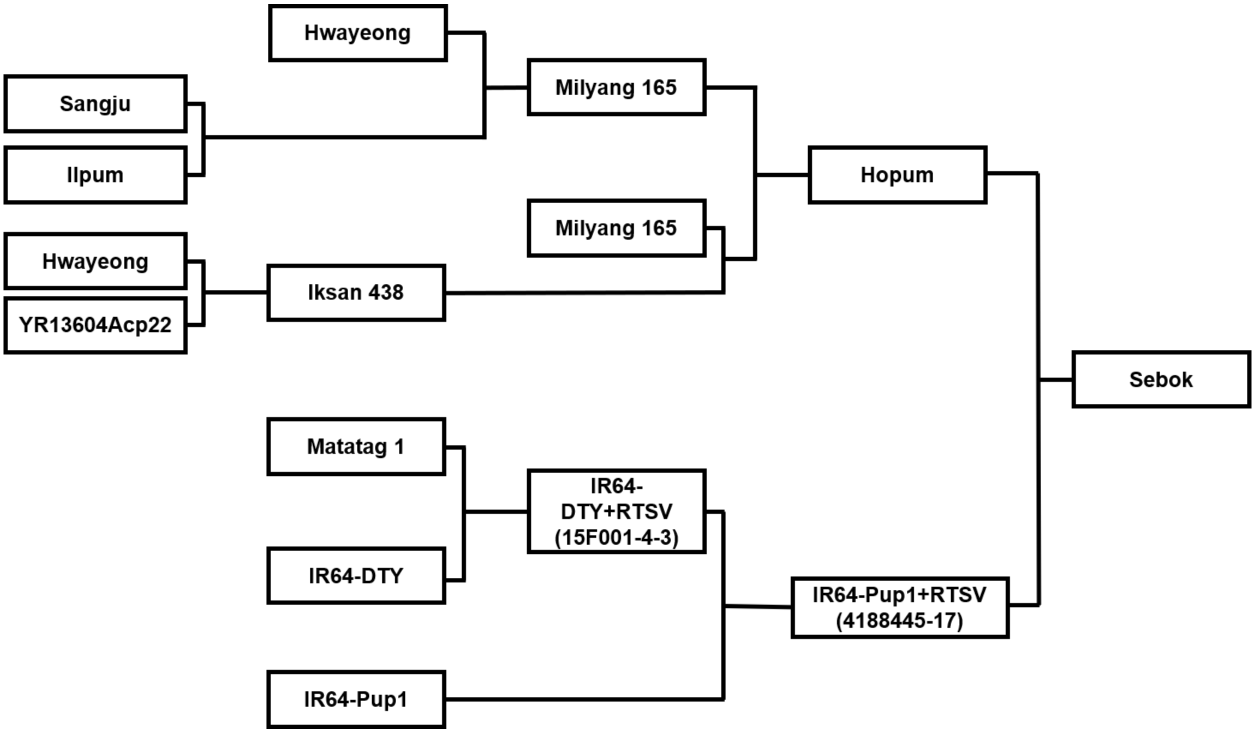

Note: To establish high-quality and multi-stress resistant rice varieties, the desirable phosphorus uptake 1 (*Pup1*), quantitative trait locus (QTL), and rice tungro virus resistance (*RTSV*) genes were introduced into Hopum, an elite *Japonica* variety known for its superior quality. The breeding approach involved several key steps. First, a promising individual, 15F1001-4-3, was selected from the F_3 population derived from the cross between IR64-DTY (GID 4537776) and Matatag 1 (GID 4537724). Next, this individual was crossed with IR64-Pup1 (GID 4537756). Marker-assisted selection was used to identify individuals carrying the *Pup1* and *RTSV* genes. During the development of rice breeding lines, a colored line was observed in the dehulled rice. Further analysis revealed higher levels of polyphenols and flavonoids compared with Hopum. In BC_2F_7, a rice variety known as 'Sebok' was developed.

Figure S2. Standard curve of ascorbic acid (vitamin C).

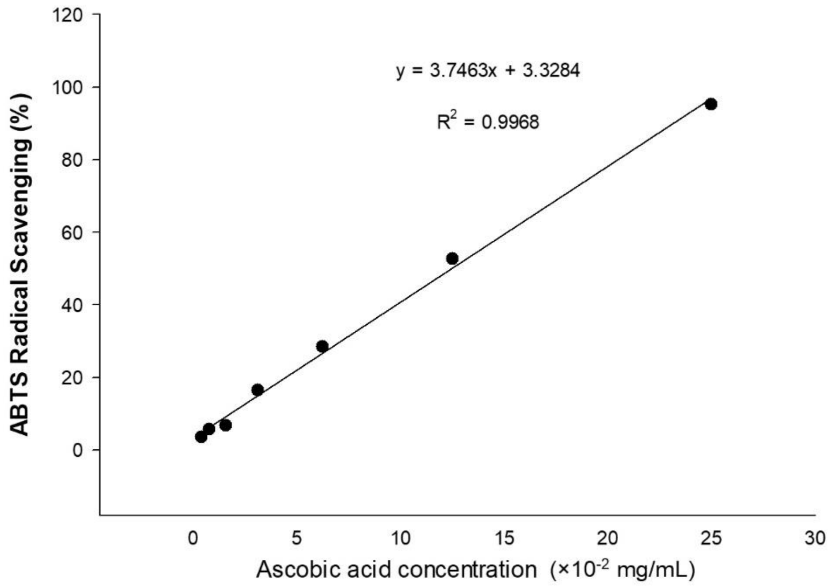

Note: The vitamin C standard curve was linear across a concentration range of 3.9065–250 μg/mL. The regression equation is "y = 3.7463x + 3.3284" (R^2 = 0.9968); where y is the scavenging activity (%) value.

Figure S3. Calibration curve of tannic acid using for total polyphenols content evaluation

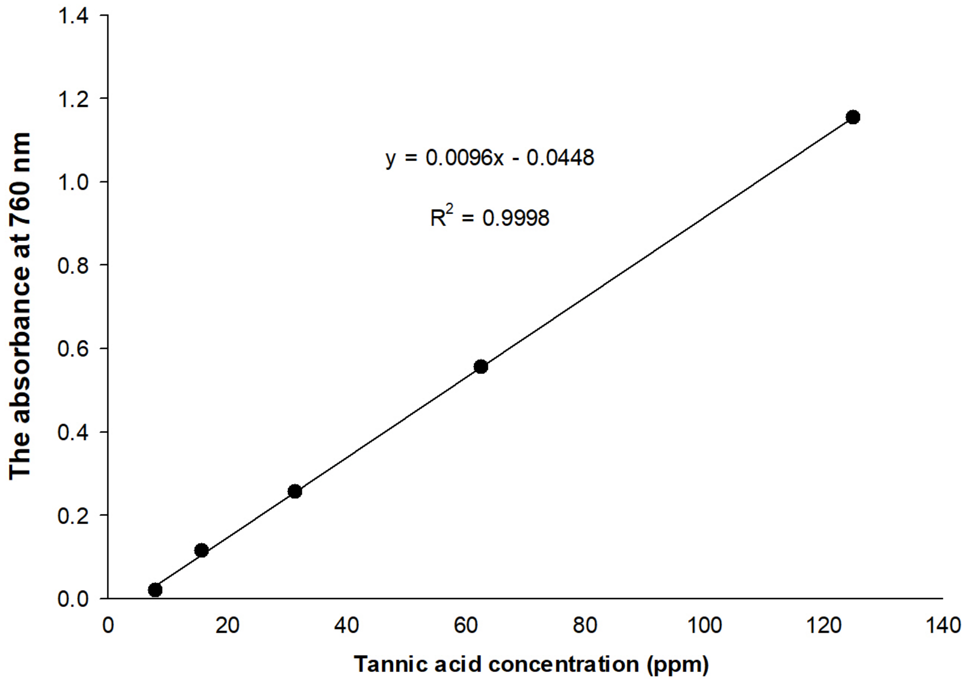

Figure S4. Calibration curve of quercetin using for total flavonoids content evaluation

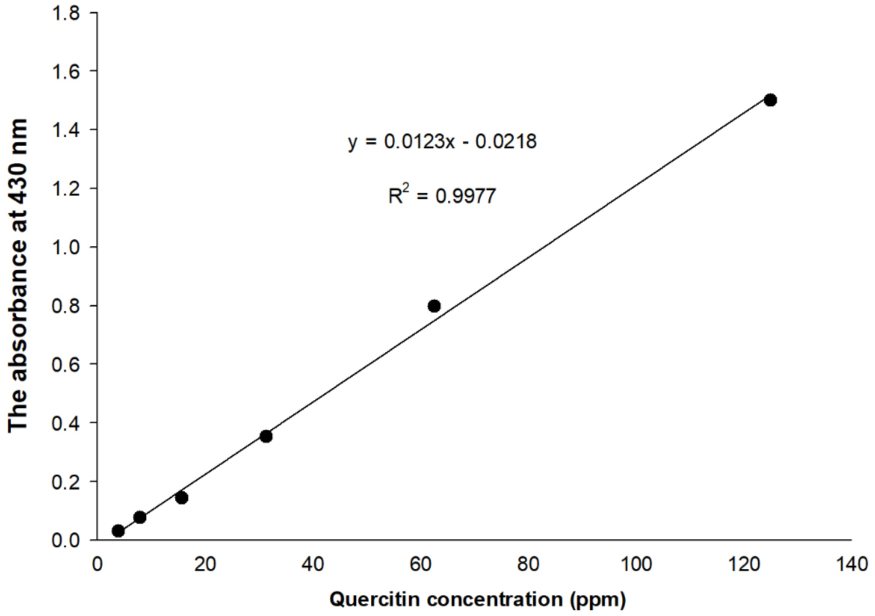

Table S1. Differentiated melan-a cell scoring

Benchmark	Score
Cell size lower than 50 µm and pigmentation lower than 50% of cytoplasmic area with a low distribution.	1+
Cell size lower than 50 µm and pigmentation equal or higher than 50% of cytoplasmic area with a higher distribution.	2+
Cell size larger than 51 µm and pigmentation throughout the cytoplasmic area.	3+
Cell size larger than 51 µm and pigmentation densely packed throughout the cytoplasmic area.	4+
